# Supplementary material for: Mutations in PCYT2 disrupt etherlipid biosynthesis and cause a complex hereditary spastic paraplegia
Source: Brain. 2019 Oct 22;142(11):3382–97. doi: 10.1093/brain/awz291 (PMC6821184; doi:10.1093/brain/awz291)
Supplement: awz291_Supplementary_Data [file awz291_supplementary_data.zip › awz291-Suppl_data/Supplementary_Data4.zip!/Supplementary information 3 - fibroblast lipidomics.zip!/Fibroblast lipidomics/Controle vs Patient2/Statistical plots and data/PC[O] species/Volcano plot_alternative.pdf]

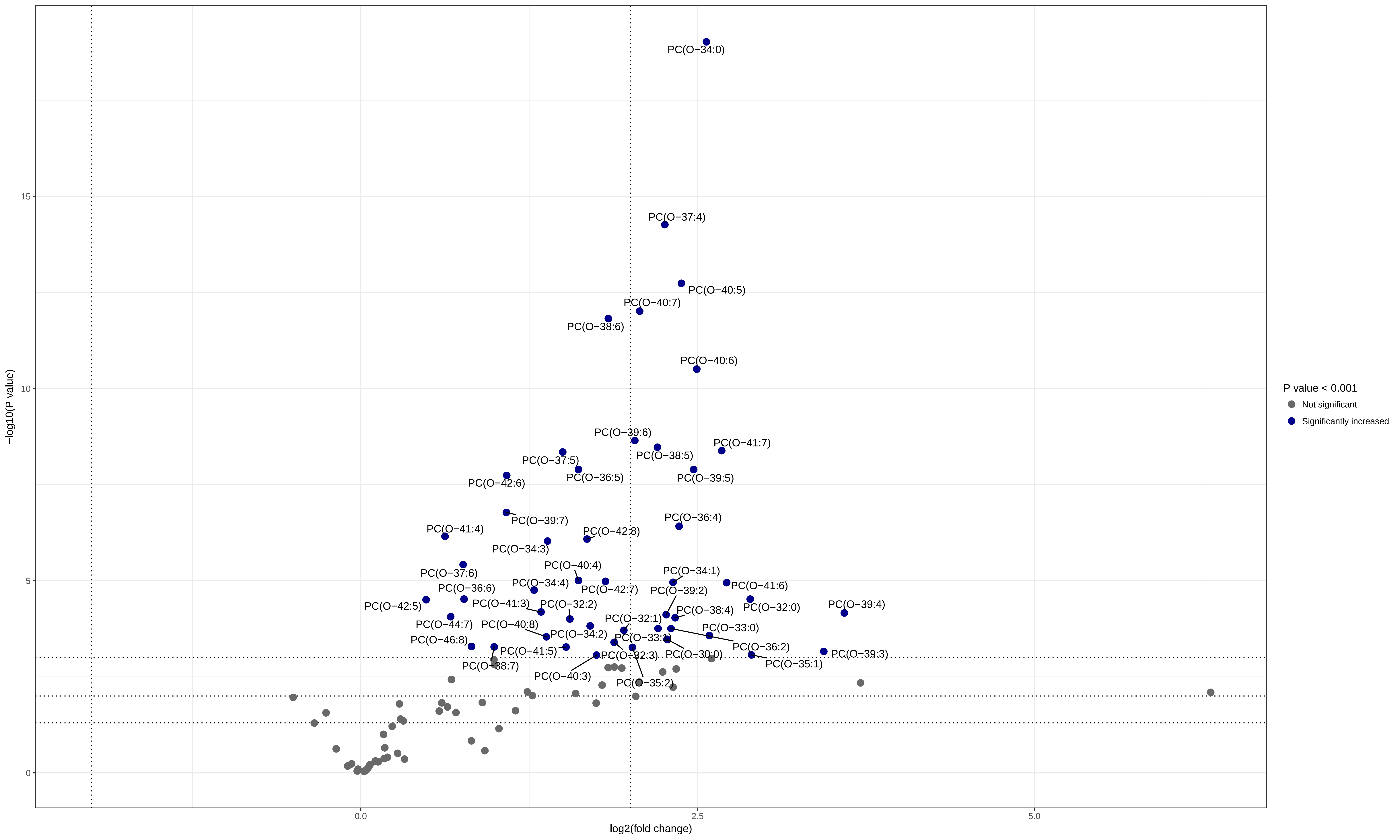

P value < 0.001
Not significant
Significantly increased
−log10(P value)
log2(fold change)
0
5
10
15
0.0
2.5
5.0
PC(O−34:0)
PC(O−37:4)
PC(O−40:5)
PC(O−40:7)
PC(O−38:6)
PC(O−40:6)
PC(O−39:6)
PC(O−41:7)
PC(O−38:5)
PC(O−37:5)
PC(O−36:5)
PC(O−39:5)
PC(O−42:6)
PC(O−39:7)
PC(O−36:4)
PC(O−41:4)
PC(O−42:8)
PC(O−34:3)
PC(O−40:4)
PC(O−34:1)
PC(O−37:6)
PC(O−34:4)
PC(O−41:6)
PC(O−36:6)
PC(O−42:7)
PC(O−39:2)
PC(O−42:5)
PC(O−41:3)
PC(O−32:2)
PC(O−32:0)
PC(O−39:4)
PC(O−38:4)
PC(O−44:7)
PC(O−40:8)
PC(O−32:1)
PC(O−33:0)
PC(O−34:2)
PC(O−46:8)
PC(O−33:1)
PC(O−36:2)
PC(O−41:5)
PC(O−32:3)
PC(O−30:0)
PC(O−39:3)
PC(O−38:7)
PC(O−35:1)
PC(O−40:3)
PC(O−35:2)
